# Mechanism of Protein Kinetic Stabilization by Engineered Disulfide Crosslinks

**DOI:** 10.1371/journal.pone.0070013

**Published:** 2013-07-30

**Authors:** Inmaculada Sanchez-Romero, Antonio Ariza, Keith S. Wilson, Michael Skjøt, Jesper Vind, Leonardo De Maria, Lars K. Skov, Jose M. Sanchez-Ruiz

**Affiliations:** 1 Facultad de Ciencias, Departamento de Quimica Fisica, Universidad de Granada, Granada, Spain; 2 Structural Biology Laboratory, Department of Chemistry, University of York, Heslington, York, United Kingdom; 3 Novozymes A/S, Bagsværd, Denmark; Consejo Superior de Investigaciones Cientificas, Spain

## Abstract

The impact of disulfide bonds on protein stability goes beyond simple equilibrium thermodynamics effects associated with the conformational entropy of the unfolded state. Indeed, disulfide crosslinks may play a role in the prevention of dysfunctional association and strongly affect the rates of irreversible enzyme inactivation, highly relevant in biotechnological applications. While these kinetic-stability effects remain poorly understood, by analogy with proposed mechanisms for processes of protein aggregation and fibrillogenesis, we propose that they may be determined by the properties of sparsely-populated, partially-unfolded intermediates. Here we report the successful design, on the basis of high temperature molecular-dynamics simulations, of six thermodynamically and kinetically stabilized variants of phytase from *Citrobacter braakii* (a biotechnologically important enzyme) with one, two or three engineered disulfides. Activity measurements and 3D crystal structure determination demonstrate that the engineered crosslinks do not cause dramatic alterations in the native structure. The inactivation kinetics for all the variants displays a strongly non-Arrhenius temperature dependence, with the time-scale for the irreversible denaturation process reaching a minimum at a given temperature within the range of the denaturation transition. We show this striking feature to be a signature of a key role played by a partially unfolded, intermediate state/ensemble. Energetic and mutational analyses confirm that the intermediate is highly unfolded (akin to a proposed critical intermediate in the misfolding of the prion protein), a result that explains the observed kinetic stabilization. Our results provide a rationale for the kinetic-stability consequences of disulfide-crosslink engineering and an experimental methodology to arrive at energetic/structural descriptions of the sparsely populated and elusive intermediates that play key roles in irreversible protein denaturation.

## Introduction

Natural disulfide bonds have been known for many years to contribute to native protein stabilization [1]. In the context of a two-state (native to unfolded) equilibrium denaturation process, disulfide-bond stabilization is often viewed as a consequence of the decrease in the conformational entropy of the unfolded state caused by the presence of the crosslink [2], [3]. Hence, it is generally accepted that engineering disulfide bridges is an efficient way to stabilize proteins, provided that they are introduced at locations in which they do not distort or strain the native fold or perturb the active site [4], [5], [6], [7], [8], [9], [10], [11].

However, there is a fundamental difference between a protein’s thermodynamic and kinetic stability and clearly disulfide bridges may also have an important effect on the latter. Indeed, in a biotechnological setting, the rate of irreversible enzyme inactivation may well be affected by the presence of disulfide bridges and be more relevant than the unfolding free energy change [12], [13]. In addition, disulfide bridges in aggregation prone regions can help prevent the dysfunctional association of proteins [14], [15]. While thermodynamic stability is related to the free energy difference between the native and the unfolded states, in the context of transition-state theory, kinetic stability is determined by that between the native state and the transition states for the rate limiting steps in the irreversible denaturation pathway [16]. Furthermore, the mechanisms of irreversible denaturation can be exceedingly complex [17], [18], [19], with non-native intermediate species (states, ensembles) playing a fundamental role. Accordingly, while a correspondence between disulfide bridge effects on thermodynamic and kinetic stability cannot be postulated *a priori*, very few experimental studies have addressed the relative importance of these effects. For instance, the failure of designed disulfide bridges to stabilize some proteins has been related to the irreversible, kinetically-controlled nature of the denaturation process [20]. However, this interpretation remains unproven, given the low level of success often met in the rational design of stabilizing disulfide bridges [21], [22].

We here use rational computation-based procedures to design stabilizing disulfide bridges in the phytase from *Citrobacter braakii*, a biotechnologically important enzyme currently used in RONOZYME HiPhos (DSM Nutritional Products, Basel, Switzerland). The design procedure, based on heated Molecular Dynamics simulations, was highly successful and allowed us to prepare several stabilized variants with 1, 2 or 3 engineered bridges. Differential scanning calorimetry combined with extensive thermal inactivation reveals the main features of the effect of the bridges on thermodynamic stability and their impact on kinetic stability. These variants thus provide an excellent comparison of the effects of engineered disulfide bridges on thermodynamic and kinetic stability. We report the crystal structure of one of the variants.

## Results and Discussions

### Computational Design of Disulfide Bridges

**Figure 1:**
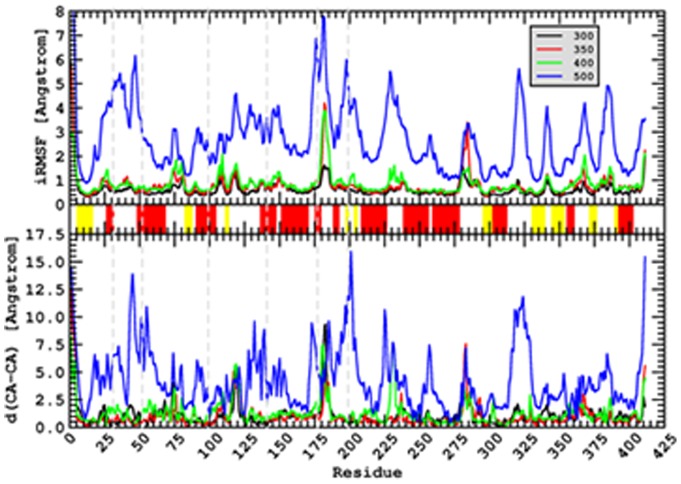
Molecular Dynamics simulations performed on a homology model of the phytase from *C. braakii*. Simulations were performed at several temperatures (shown in Kelvin in the inset of the upper panel). Upper panel: isotropic root mean square deviations (iRMSF) of the Cα at the different temperatures. Middle panel: secondary structure assignment with red regions representing α-helices and yellow regions representing β-sheets. Bottom panel: mean distances between Cα of the starting structure and Cα of the structures at different temperatures. These profiles suggest that at high temperature the enzyme displays an unfolding behavior while at low temperatures the available thermal energy is only able to excite fluctuations of specific regions. Dashed grey lines mark the position of the residues mutated to engineer disulfide bridges.

The design of the engineered disulfide bridges was based on molecular dynamics simulations performed at several temperatures on a homology model of the phytase from *C. braakii*, no 3D structure being available at that time (see Text S1 for details). Molecular dynamics simulations are well established for the study of protein folding and unfolding [23], [24], [25] and, indeed, the isotropic root mean square deviations (iRMSF) of the Cα carbons at the different temperatures suggest that the enzyme displays a clear unfolding behavior in the simulations performed at 500 K (see Figure 1) while at the lower temperatures the available thermal energy is only able to excite fluctuations of specific regions.

**Figure 2:**
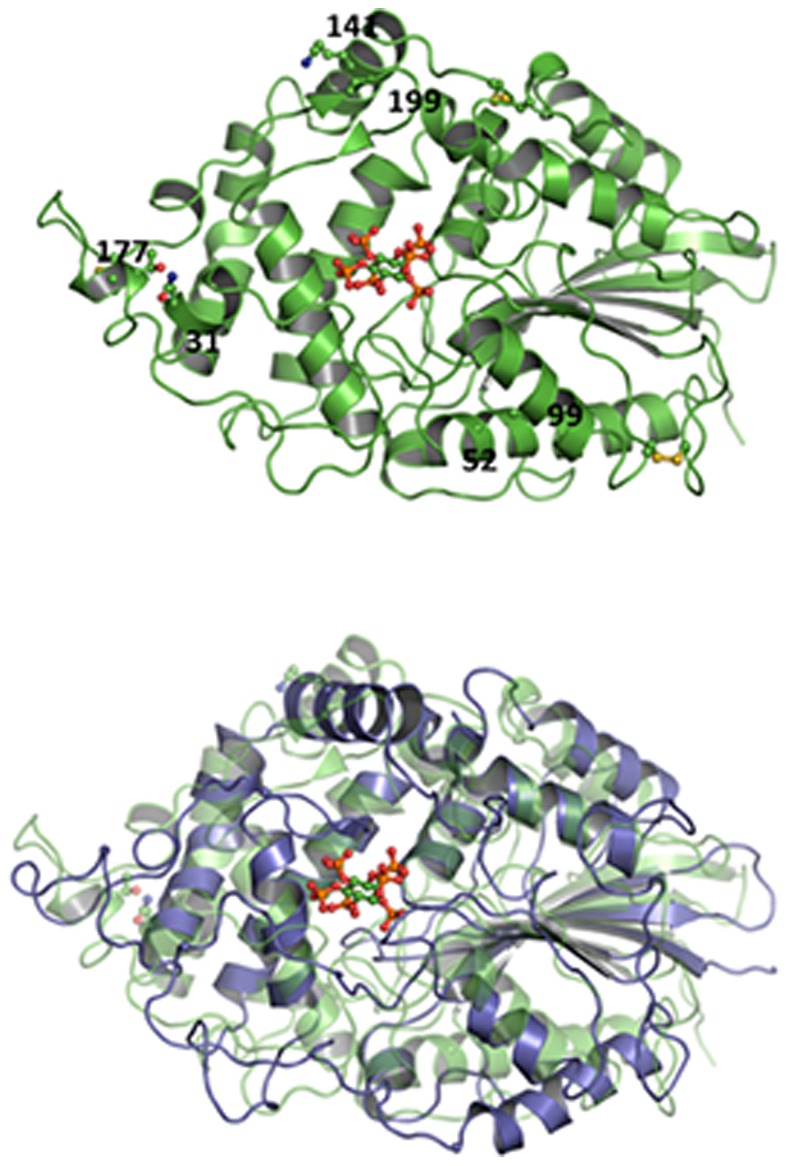
Disulfide crosslink design. Upper panel: structure of the homology model of the phytase from *C. braakii* used in Molecular Dynamics simulations showing the residues mutated to introduce disulfide bridges. Bottom panel: superposition of the MD structures at 500 K and low temperature (blue and green, respectively). Regions targeted for disulfide bridge engineering are those showing significant displacement between the two structures.

The design of the additional disulfide bridges targeted regions of the structure with a substantial tendency to unfold in the Molecular Dynamics simulations. The strategy was to introduce the disulfides in regions of the structure significantly displaced with respect to one another at 500 K, in comparison with the starting structure (Figure 2). Once these regions were identified, the positions to mutate were chosen by geometric considerations in the protein structure. This approach was based on the following hypotheses: i) the targeted regions are likely to be more flexible and may be expected to accommodate the engineered disulfides without straining or distorting the native structure; ii) the targeted regions could be unfolded in the kinetically relevant transition state and their stabilization by disulfide crosslinking may increase the free-energy barrier for irreversible denaturation and enhance kinetic stability. Since we aimed at enhancing both the thermodynamic and kinetic stabilities of the phytase without compromising function, we deemed it advisable to focus engineering on those regions with a substantial tendency to unfold which lined the rim of the substrate binding pocket or were in direct contact with them, involving key residues for the enzymatic activity.

The procedure described above led us to prepare three variants (see Text S1 for details) each with a single engineered disulfide bridge: S1 (N31C/T177C), S2 (G52C/A99C) and S3 (K141C/V199C). Among these three single disulfide variants, S2 proved to be the most stable (see below). Therefore, we deemed it appropriate also to study multiple-disulfide variants in which the bridge in S2 (G52C/A99C) was combined with the other bridges. The following variants with two or three of these bridges were therefore made: D1 (N31C/T177C+G52C/A99C), D2 (G52C/A99C+K141C/V199C) and T (N31C/T177C+G52C/A99C+K141C/V199C).

### Preliminary Assessment of the Activity and Stability of the Variants with Engineered Disulfide Bridges

**Figure 3:**
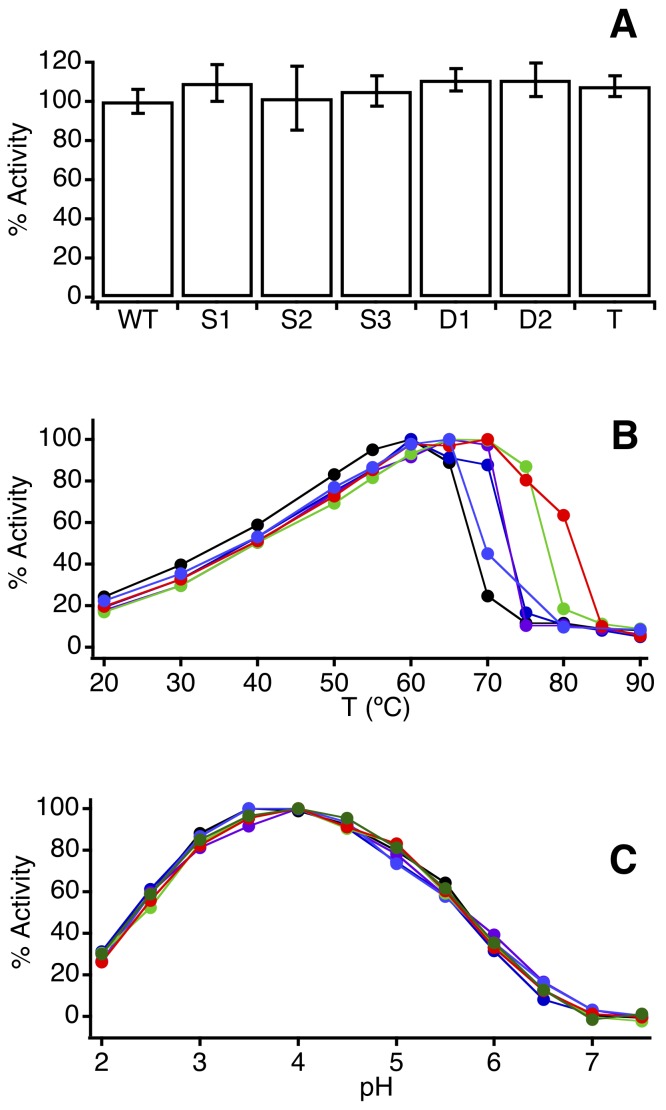
Preliminary assessment of the activity and stability of the phytase variants with engineered disulfide bridges. (A) Activity values for the wild-type and the variants studied at pH 4.5. (B) Profiles of activity vs. temperature at pH 4.5. The drop in activity at the higher temperatures indicates denaturation and provides a first estimate of the thermal stability. (C) Profiles of activity vs. pH for wild-type and the variants. In panels B and C, the maximum activity value of each profile is normalized to 100, while in panel A wild-type phytase is assigned a value of 100. Code color for the variants in panels B and C refers to the number of engineered bridges (black 0, blue 1, green 2, red 3) and is more clearly apparent in Figure 5A.

All the variants showed similar activity levels in the standard phytase assay (Figure 3 A; see Text S1 for details) and essentially identical pH profiles (Figure 3 B). Furthermore, very similar temperature dependencies of activity are observed at sub-denaturational temperatures (Figure 3 C) while the high temperature activity drop associated with irreversible denaturation is shifted to higher temperatures as the number of disulfide bridges increases. These results suggest that, while the engineered crosslinks do enhance stability, they do not cause dramatic alterations in the native, functional structure of the enzyme, corroborated by the 3D-structure of one of the variants. Dynamic light scattering experiments support that irreversible denaturation in this system is linked to significant protein aggregation (Figure S1).

### 3D-structure of a Phytase Variant

**Figure 4:**
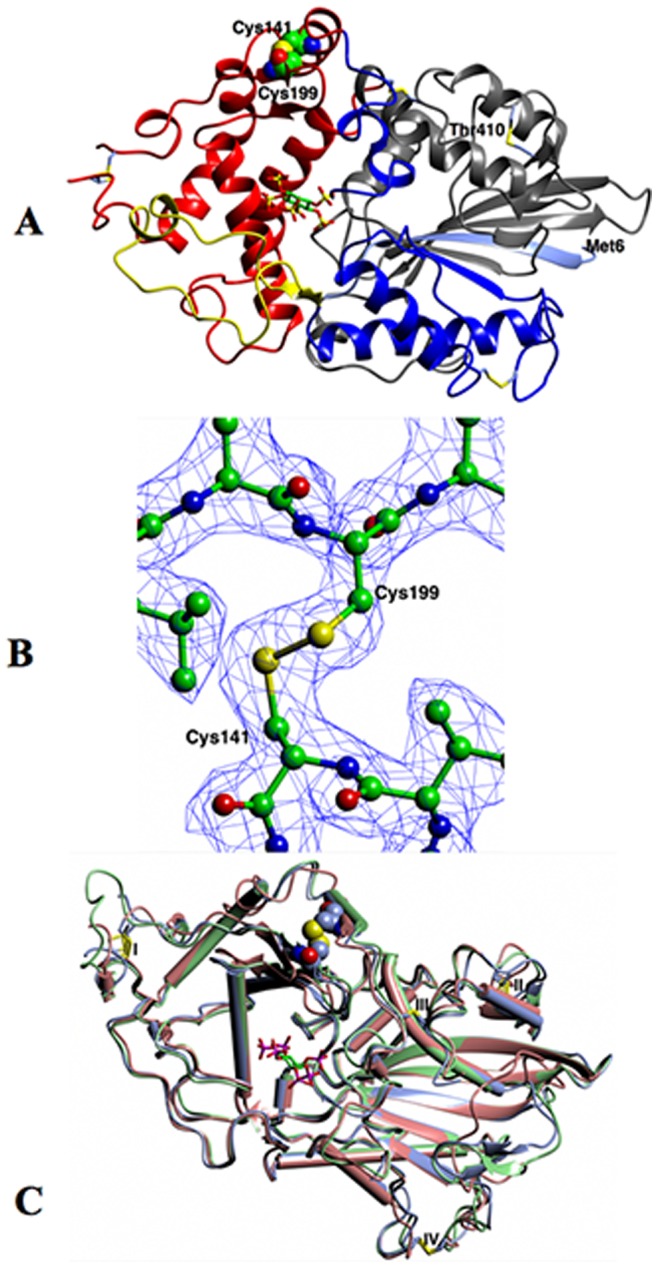
Structural consequences of an engineered disulfide crosslink. (A) The fold of Chain A of *C. braakii* phytase in ribbon format, with residues 6–18 in ice blue, 19–46 yellow, 47–135 blue, 136–259 red and 260–410 grey. The four conserved disulfide bridges are shown as cylinders, and the engineered ones as spheres. The phytate analogue myo-inositol hexakissulfate – shown as cylinders-has been modeled into the active site based on its position in its complex with *H. alvei* phytase (PDB 4aro). (B) The electron density in the final 2F_o_–F_c_ synthesis contoured at the 1σ level around the engineered disulfide bridge between residues 141 and 199. (C) Superposition of the structures of the *E. coli* (green, PDB 1dkq) and *H. alvei* (coral, PDB 4ars) enzymes on *C. braakii* phytase (blue), using the SSM option [44] in CCP4mg. The structures are shown in worm and tube format. Figures 4 A–C were all made with CCP4mg [45].

The structure with a disulfide bridge engineered between residues 141 and 199 was refined to 2.3 Å spacing with two independent protein monomers in the asymmetric unit (see Text S1 for details). The fold (Figure 4) has two domains and is very similar to those of previous 3- and 6-phytases in the PDB from the bacteria *E. coli* [*26*] *, Hafnia alvei* [*27*], *Klebsiella pneumoniae* [*28*] and *Yersinia kristensenii* [*27*] and the fungi *Aspergillus ficuum* [*29*], *A. fumigatus* [*30*], *A. niger* [*31*] and *Debaryomyces castellii* [*32*] and is typical of members of the histidine acid phosphatase superfamily. The major domain on the right is composed of residues 6–28, 47–135 and 260–410 and is colored blue and grey. At its backbone is a mainly parallel β-sheet, surrounded by α-helices and loops. The second domain, primarily α-helical, is composed of two insertions (residues 39–46 and 136–259) in the first domain and is seen on the left of the Figure in red/yellow. The active site is at the interface between the two domains, its position indicated in the Figure by superimposing the *myo*-inositol hexakissulfate complex of the *H. alvei* [*27*] enzyme (PDB 4aro, 4arv and 4aru). The engineered disulfide between residues 141 and 199 crosslinks an α-helix and a coiled loop of the α-helical domain and has well-defined electron density (Figure 4 B) indicating a highly successful introduction of this new element into the phytase fold.

The high degree of conservation of fold in the 3- and 6-Phytases of the HAP superfamily is illustrated by the superposition of the *C. braakii* structure on that of the *H. alvei* enzyme in Figure 4B. The rmsd in ∼380 equivalent Cα positions is 1.18 Å. The four disulfide bridges can be seen to be totally conserved.

### Determination of Termal Stability Through Differential Scanning Calorimetry

**Figure 5:**
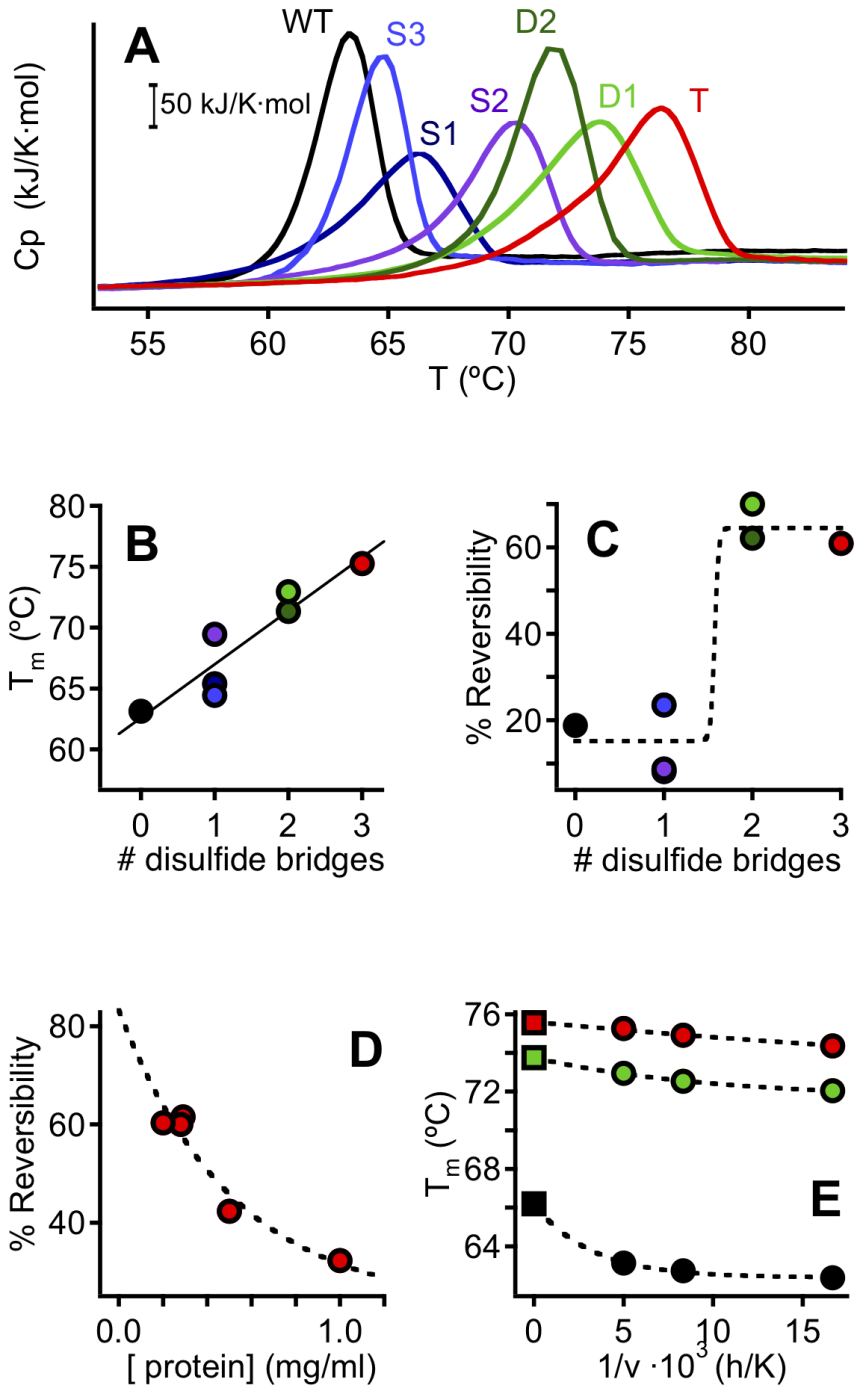
Differential scanning calorimetry (DSC) of the thermal denaturation of wild-type phytase and the disulfide variants. A) Representative DSC profiles at a protein concentration of 0.5 mg/mL and a scan rate of 200 degrees/hour. B) Plot of transition temperature (derived from the results in panel A) versus number of engineered bridges. C) Plot of degree of reversibility of the thermal denaturation process versus the number of engineered bridges. The degrees of reversibility shown were derived from DSC experiments performed with a protein concentration of 0.25 mg/mL, at a scanning rate of 200°C /hour. In all cases, the first scan was stopped at 100°C and a re-heating run was performed after letting the sample cool in the calorimetric cell. The degree of reversibility was calculated as the ratio between the maximum heat capacity values for the reheating and the first transitions after suitable chemical baseline correction. D) Degree of reversibility for the variant with three engineered bridges versus protein concentration. E) Scan-rate effect on the transition temperatures (circles) derived from DSC experiments. Extrapolation to 1/v = 0 (i.e., to infinite scan-rate) should remove kinetic distortions associated to partial irreversibility (the squares at 1/v = 0 actually represent the denaturation temperatures for equilibrium unfolding determined from model fitting to rate data). In panels B–E the colors refer to the different variant studied as specified in panel A.

DSC transitions (Figure 5: see Text S1 for details) for the thermal denaturation of wild-type phytase and the engineered variants were only partially reversible and showed a small but significant scan-rate dependence (Figure 5E). This indicates that the thermally-induced transitions reflect a degree of irreversible denaturation and are probably kinetically distorted by the process responsible for the irreversibility. We have therefore refrained from carrying out a detailed equilibrium thermodynamic analysis of the transitions. However, some general and robust trends are evident. Firstly, the transition temperature increases monotonically with the number of engineered bridges (Figure 5B), which clearly validates the approach used for computational disulfide design. Indeed, the effects of the engineered disulfides on thermal stability appear to be roughly additive; for instance adding the T_m_ enhancements (relative to wt) found for the single variants (2.2, 6.3 and 1.3°C for S1, S2 and S3, respectively), ΔT_m_ values of 8.5, 7.6 and 9.8°C are predicted for D1, D2 and T, while the experimental T_m_ enhancements relative to wt for these variants are 9.8 (D1), 8.2 (D2) and 12.1°C (T). Secondly, the reversibility is higher for variants with two and three engineered bridges (Figure 5C). In addition, the degree of reversibility decreases with increasing protein concentration (Figure 5D), suggesting a role for aggregation in the irreversible denaturation, an interpretation supported by dynamic light scattering experiments (Figure S1).

### Thermal Inactivation Kinetics

Irreversible denaturation of wild-type phytase and the variants was characterized by thermal inactivation kinetics. Briefly, samples were kept at a given temperature, aliquots were withdrawn at several times, quickly cooled down to 0°C and assayed for phytase activity (see Text S1 for details). Unfolded phytase (as well as partially-unfolded states “reversibly-linked” to the native state) should be able to fold to the native, active protein upon cooling, so the observed fall in activity with time can be primarily attributed to irreversible denaturation.

**Figure 6:**
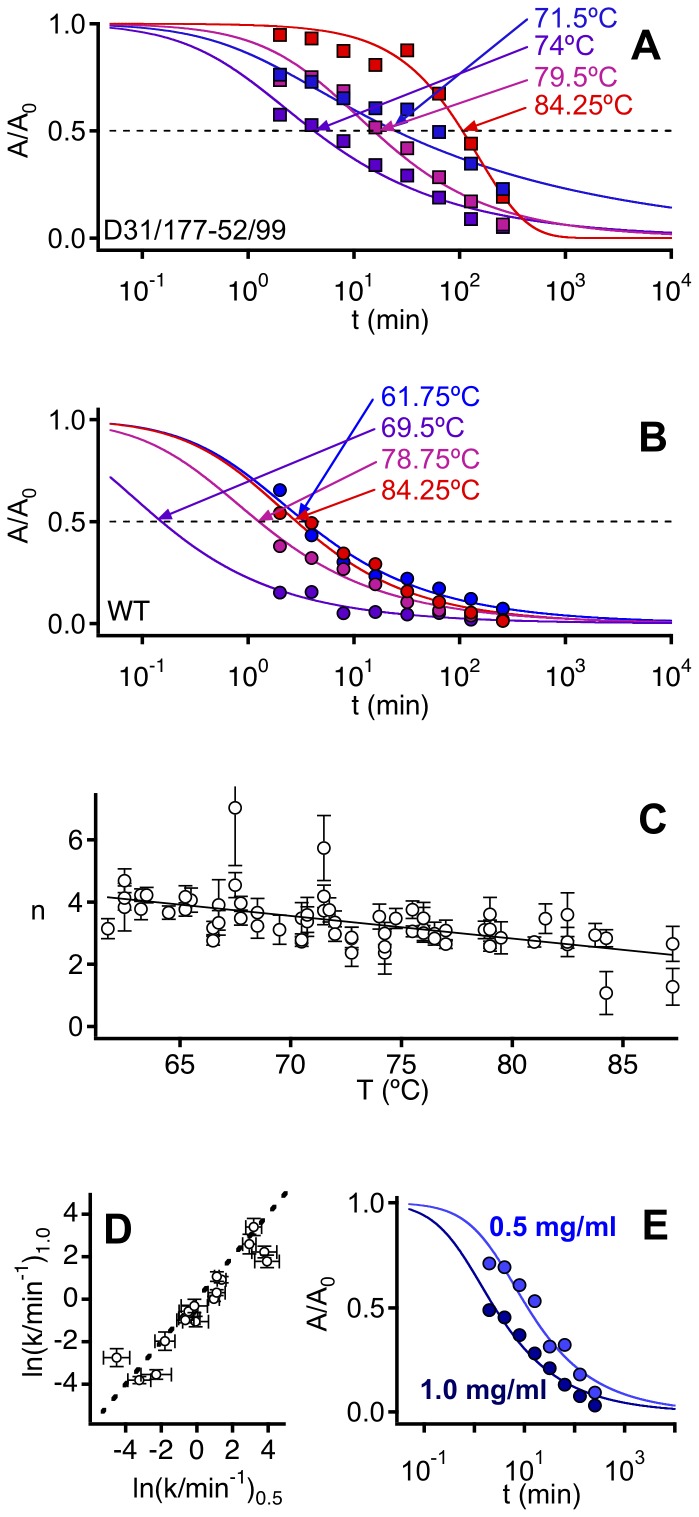
Thermal inactivation kinetics data for wild-type phytase and variants. (A) and (B) Illustrative plots of activity versus time for experiments performed at several temperatures with (A) a variant with two engineered bridges and (B) wild-type. Total protein concentration is 0.5 mg/mL. The continuous lines represent the best fits of equation 1 to the experimental data. The fastest kinetics are observed at an intermediate temperature (74°C in A and 69.5°C in (B). (C) Values of the reaction order derived from the fitting of equation 1 to inactivation profiles for wild-type phytase and all the variants. (D) Agreement between the rate constants obtained with total protein concentration of 0.5 and 1 mg/mL. (E) Representative example of the protein concentration effect on the rate of irreversible denaturation (variant D1, 76°C).

Thermal inactivation profiles were determined for each variant (representative examples in Figure 6) at several temperatures and total protein concentrations. For any given temperature, kinetic profiles were found to depend on total protein concentration, again consistent with a role for aggregation in the irreversible denaturation. However, mechanisms of protein aggregation can be exceedingly complex, involving conformational re-arrangement, nucleation and growth steps, as well as processes of aggregate fragmentation, aggregate coalescence to yield larger aggregates and phase separation of insoluble aggregates [33], [34]. Analysis of typically featureless thermal inactivation profiles, such as those in Figure 6, in terms of a detailed mechanism is clearly not possible. Our approach is more modest but more robust. We aim at a simple, approximate phenomenological description of the profiles so that a single metric for the time scale of the irreversible denaturation process can be derived. Since no lag phases were observed in the experimental profiles, we used a simple n-order chemical kinetics equation: 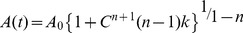
(1)where *A*(*t*) and *A_0_* stand for the activities at time *t* and time zero, *C* is the total protein concentration and *k* is the *n*-order rate constant for the process. Fits of equation 1 to the thermal inactivation profiles were visually good (Figures 6 A and 6 B) and yielded *n* values typically of about 3–4 (Figure 6 C), a result generally consistent with the proposed role of aggregation in phytase irreversible denaturation. The phenomenological adequacy of equation 1 within the 0.5–1 mg/mL range of total protein concentration is further supported by the agreement (Figures 6D,E) of the rate constants obtained at these two protein concentrations (equation 1 possibly breaks down at lower protein concentrations: see Figure S2).

This allows a suitable metric of the time scale for irreversible denaturation to be calculated as the time τ_1/2_ at which the activity falls to half the initial value: 
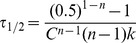
(2)


All our subsequent analyses are based upon the temperature and mutational effects on this time scale.

### Analysis of the Mutation and Temperature Dependencies of the Time Scale for Irreversible Denaturation

**Figure 7:**
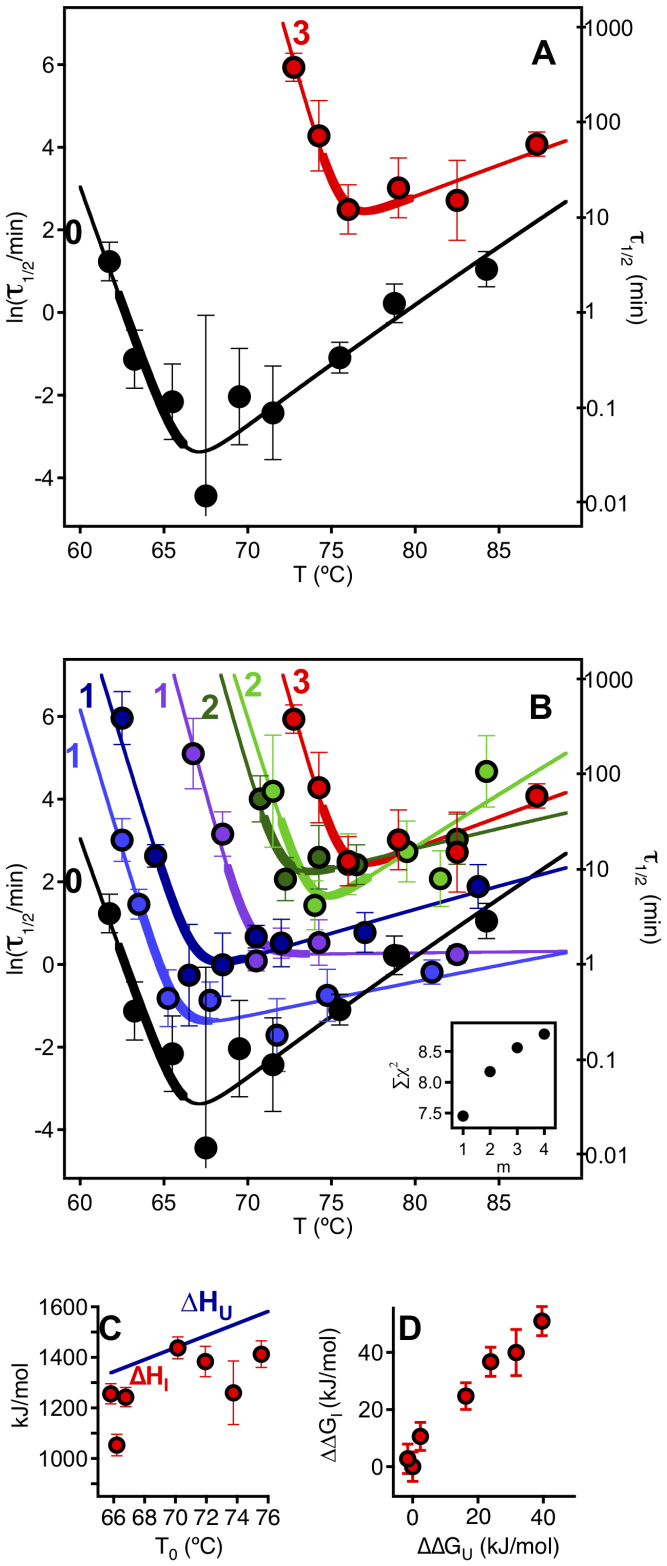
Temperature dependence of the time scale (τ_1/2_) for the irreversible denaturation of wild type phytase and variants. (A) and (B) Plots of ln(τ_1/2_) versus temperature for the variants (for the sake of clarity, only wild type and the variant with three engineered bridges are included in panel A). The continuous lines represent the best fits of equation 3 and the Inset in panel B is a plot of a measure of goodness of fit (χ^2^) versus the reaction order m in equation 3 (see main text and Text S2 for details). Thicker lines indicate the temperature range of denaturation transition as seen by DSC (Figure 5A). The colors of the lines and data points refer to the variant, as specified in panel A of Figure 5, with the number of engineered bridges indicated. (C) Enthalpies of the unfolded and intermediate states (relative to the native state) plotted versus the corresponding T_0_ value. (D) Correlation between the effect of engineered disulfide bridges on the free energy of the unfolded and intermediate states (relative to the native state) at a temperature of 70°C.

**Figure 8:**
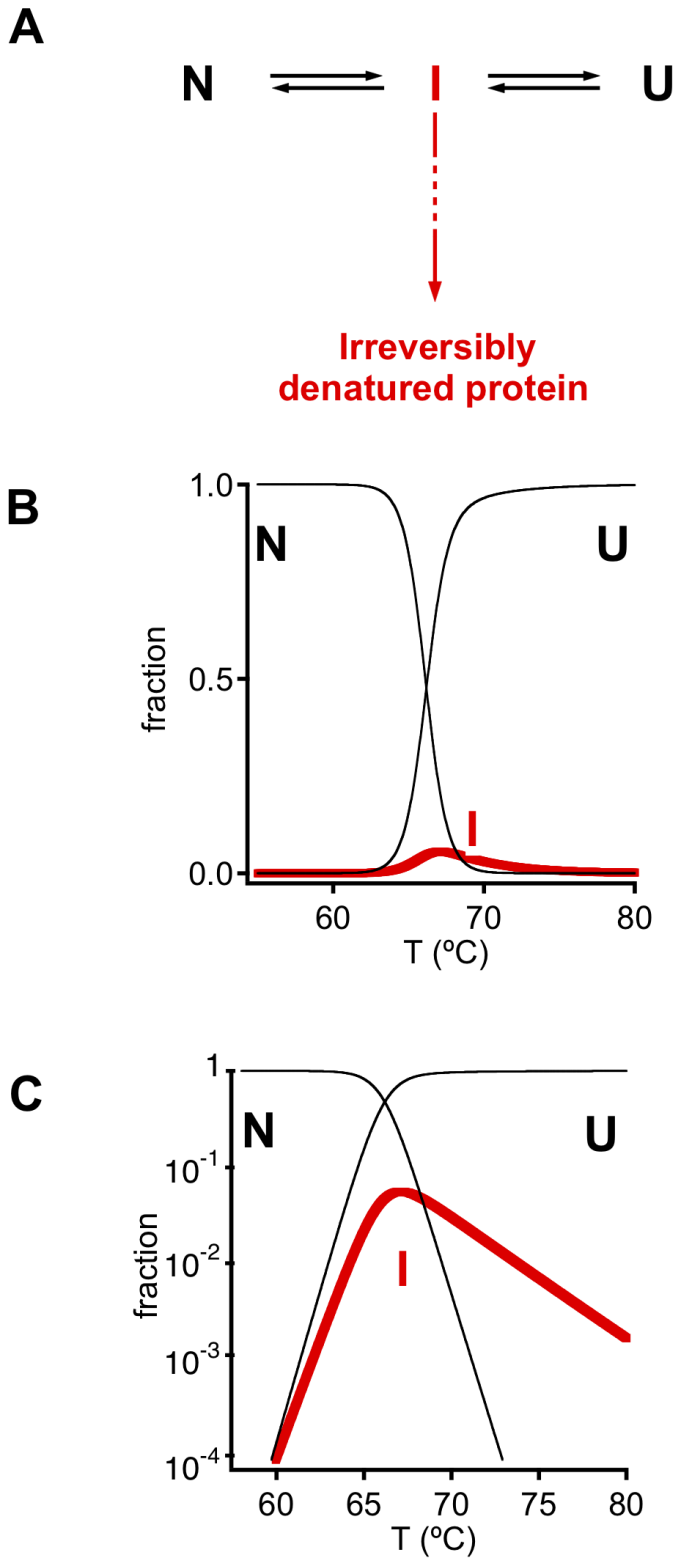
Model used to derive equation 3 and describe the experimental thermal inactivation profiles for wild-type phytase and variants (Figures 7A and B). (A) An intermediate state (or ensemble) is assumed to be critical for the irreversible denaturation process. (B) and (C) At equilibrium, the population of I is always low, although it reaches a maximum roughly within the temperature range of the transition. When using a logarithmic scale (panel C) the shape of the population of I versus temperature profiles matches that of the ln(τ_1/2_) versus temperature plots of Figure 7, with the maximum of population of I corresponding to the minimum of τ_1/2_ (see **equation 3**). The profiles in panels B and C have been calculated using equations provided in Text S2.

Figures 7A and 7B show plots of time scale for irreversible denaturation versus temperature for a total protein concentration of 0.5 mg/mL (similar plots are obtained at 1 mg/mL: see Figure S3). Figure 7 shows that, for a given temperature, irreversible denaturation becomes slower (larger value of τ_1/2_) as the number of engineered disulfides is increased. However, the most surprising result is the unexpected temperature-dependence of the rate of irreversible denaturation: for essentially all variants the plot of ln(τ_1/2_) versus temperature has an asymmetric-V shape with a minimum at a temperature that roughly agrees with the temperature range of the denaturation transition seen by DSC (see figure 5A and the thicker lines in the plots of Figure 7A). This pattern strongly suggests (Figure 8) that some species, different from the native state (N) and the fully unfolded state (U) are critical for the irreversible denaturation process and that such species represent a partially-folded intermediate state (I) that attains a kinetically relevant concentration within the denaturation transition range. Straightforward theoretical analysis of this model (see Text S2) leads to a simple equation describing the temperature dependence of the time scale for irreversible denaturation: 
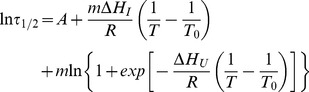
(3)where *T_0_* is the equilibrium denaturation temperature (i.e., the temperature at which the equilibrium constant for the N to U conversion is unity), A is a constant (related to the value of the equilibrium constant for the N to I conversion at *T_0_*), *m* is the reaction order for species I in the phenomenological rate equation and *ΔH_I_* and *ΔH_U_* are, respectively, the enthalpies of I and U relative to the native state. Fits of equation 3 to the experimental τ_1/2_ versus *T* data were excellent (Figure 7 and Figure S3; see Text S2 for additional details on the fitting process) and are further validated by the fact that the values obtained for *T_0_* (the equilibrium unfolding temperature) are consistent with the extrapolations to infinite scan-rate of the denaturation temperatures determined from the DSC transitions (Figure 5E).

It is important to note that our analysis of the model in Figure 8A assumes that the population of intermediate species is always much lower than the total protein concentration (see Text S2). While the species I is not assumed to be significantly populated, its critical role in kinetics is reflected in the experimental τ_1/2_ versus *T* profiles and its energetics are readily derived from the fits of equation 3 to those profiles. Thus, the enthalpy of I relative to the native state (*ΔH_I_*) is directly determined as a fitting parameter in equation 3 and a suitable metric of the free energy of I relative to N (*ΔG*′*_I_*) can be easily calculated from the values of the fitting parameters *A*, *T_0_* and *ΔH_I_* in a straightforward manner (see Text S2 for details).

The energetic description afforded by the *ΔH_I_* and *ΔG*′*_I_* values for the variants immediately indicates an extensively unfolded intermediate species, with the enthalpy of the intermediate being close to that of the unfolded state (both relative to the native state), Figure 7C. Furthermore, a plot of the free energy of the intermediate versus that of the unfolded state (both relative to the native state) including all variants studied is linear with a slope close to unity (Figure 7D). The plot of *ΔG*′*_I_* versus *ΔG_U_* is in fact a comparison between the effect of the engineered disulfide bridges on the changes in free energy for the N→I and N→U processes and, therefore, it is equivalent to a mutational φ-value analysis [35] of the structure of the intermediate. Indeed, the plot suggests a φ value around unity, consistent with a substantially unfolded intermediate species.

## Methods

See Text S1.

### Conclusions

We have used *C. braakii* phytase to probe the effect of designed disulfide bridges on protein kinetic stability. The rational design procedure, based on high temperature molecular dynamics simulations, was highly successful, leading to variants with enhanced thermodynamic stability and, furthermore, with much slower inactivation kinetics. However, as expounded below, the congruence between the effect of the engineered disulfides in the thermodynamic and kinetic stabilities is actually a complex phenomenon.

The inactivation kinetics for the variants shows a striking, non-Arrhenius temperature dependence, with the time-scale for the irreversible denaturation process generally reaching a minimum at a given temperature within the range of the unfolding transitions. This pattern is a clear signature of the key role played by an intermediate state which, being partially unfolded, becomes maximally populated at intermediate temperatures. In fact, inspired by reasoning commonly found in the literature on protein aggregation [33], we can intuitively view the “unusual” temperature dependence reported as reflecting the conformational changes required to reach the critical intermediate species. Thus the native structure needs to undergo significant unfolding to produce the key intermediate and this unfolding process becomes favored as the temperature is increased. In contrast, the unfolded state needs to become structured to reach the intermediate and such folding is disfavoured at the higher temperatures. Accordingly, the rate of the irreversible denaturation decreases with temperature when the initial state of the process is the unfolded protein, while the rate of irreversible denaturation of the native state increases with temperature. The combination of these two opposing trends produces the “V-shaped” dependencies in plots of time-scale for irreversible denaturation *versus* temperature. The “V’s” are clearly asymmetric with nearly flat high temperature branches, indicating that the intermediate is highly-unfolded retaining little structure. This interpretation is confirmed by the fittings of the experimental time-scale profiles on the basis of the model embodied in Equation 3 (Figures 7 and 8) and, in particular, by the observation that the derived enthalpy values for the intermediate species are close to those expected for the unfolded state (Figure 7C). This, of course, does not mean that the intermediate species is to be considered as roughly equivalent to the unfolded state. In fact, the intermediate species probably contains a structured region that, although necessarily small (on account of the substantially unfolded character of the intermediate), provides an efficient nucleus for the aggregation process. Indeed, since the order of reaction for the intermediate in the rate equation is approximately unity (see inset in Figure 7B) while the overall reaction order for the aggregation process is about 3–4 (Figure 6C), it appears plausible that the structured region in the intermediate is able to recruit protein molecules in other states (native and unfolded) for the aggregation process. Furthermore, the small structured region must contribute to a high free energy with respect to both the native and unfolded states to explain the low population of the intermediate and it is therefore probably non-native-like. Overall, it is clear that the intermediate species, although substantially unfolded, cannot be viewed as a “member” of the unfolded ensemble.

While many critical intermediates in protein irreversible denaturation processes (such as aggregation and fibrillogenesis) are typically described as “partially-folded”, “molten-globule-like” or even “native-like” [36], [37], [38], [39], [40], a highly-unfolded intermediate state (with at most a small structured region) has been proposed to play a key role in the misfolding of the prion protein [41], [42]. Since the key intermediate state in the thermal inactivation of phytase is also highly unfolded, we may expect mutation effects on its free energy value (measured with respect to the native state) to parallel the corresponding mutation effects on thermodynamic stability. The experimental data validate this expectation (Figure 7D).

Of course, the congruence found between the effect of the engineered disulfide bridges in the thermodynamic and kinetic stabilities is a direct consequence of the highly-unfolded character of the key intermediate in the particular case of phytase and cannot be taken as a general feature for all proteins. We can easily imagine protein systems for which the key intermediate is native-like (thermodynamic stability enhancements caused by engineered disulfide bridges will not lead to kinetic stabilization), partially unfolded (thermodynamic stability enhancements will be partially reflected in kinetic stability) or structurally polarized (only those thermodynamic stability enhancements associated to crosslinks in the region of the protein molecule that becomes unstructured in the intermediate will have consequences for kinetic stability). Nevertheless, the methodology we propose should discriminate between these alternatives. Furthermore, the usefulness of the approach goes beyond providing a general framework for the understanding of the effect of disulfide crosslinking on protein stability. While it is widely accepted that intermediate states/ensembles play key roles in many processes of protein aggregation and fibrillogenesis, their typically low population has made their characterization elusive. For instance, in the paradigmatic case of lysozyme amyloidoses, a recent characterization of the critical non-native states/ensembles relied upon sophisticated experimental methodologies combined with destabilizing solvent conditions [39], [43]. The results reported here suggest that the temperature dependencies of suitable metrics of the rate of irreversible protein denaturation may reveal distinct signatures of key intermediate states in the process and that the corresponding energetic/structural descriptions can be derived from mutational analyses of these signatures.

## Supporting Information

Figure S1 **Dynamic light scattering experiments supporting aggregation upon irreversible denaturation of phytase.**
(DOC)

Figure S2 **Rate constants obtained with total protein concentration of 0.25 and 1 mg/mL.**
(PDF)

Figure S3 **Temperature dependence of the time scale (τ1/2) for the irreversible denaturation of wild type phytase and variants at a protein concentration of 1 mg/mL.**
(PDF)

Text S1 **Experimental section.**
(DOC)

Text S2 **Theoretical analysis of a thermal inactivation model involving a critical, lowly-populated intermediate species.**
(DOC)
