# Supplementary material for: Mechanism of Protein Kinetic Stabilization by Engineered Disulfide Crosslinks
Source: PLoS One. 2013 Jul 30;8(7):e70013. doi: 10.1371/journal.pone.0070013 (PMC3728334; doi:10.1371/journal.pone.0070013)
Supplement: Figure S1 — Dynamic light scattering experiments supporting aggregation upon irreversible denaturation of phytase. (DOC) [file pone.0070013.s001.doc]

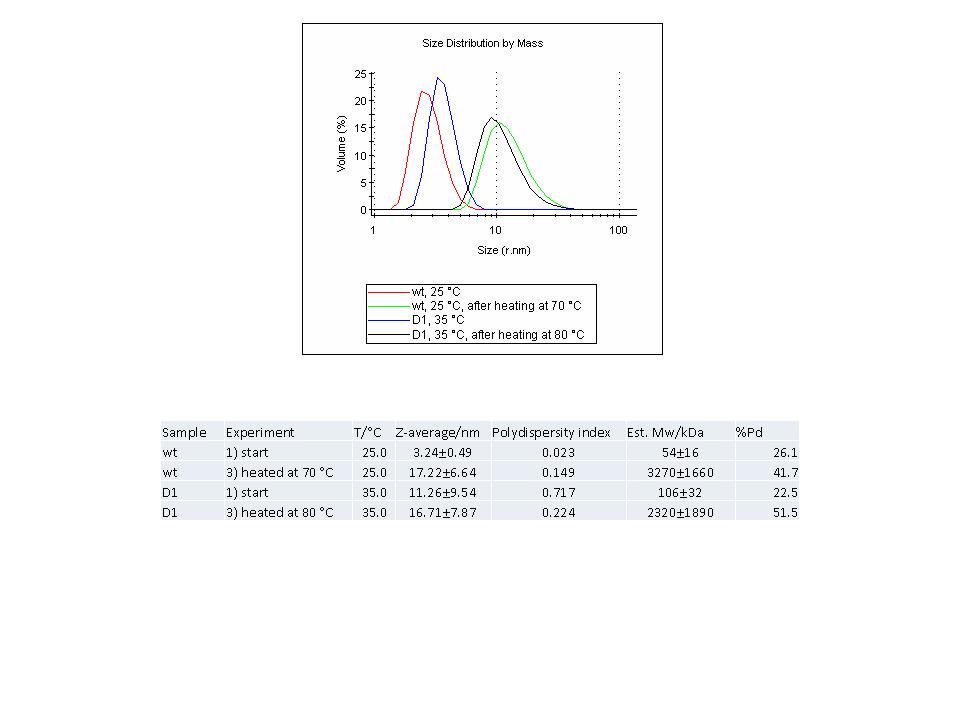


**Figure S1.** Samples of *C. braakii* phytase and variant D1 were diluted to approximately 0.5 mg/ml in 50 mM sodium acetate buffer, pH 4.5 and filtered. Dynamic light scattering (DLS) measurements were performed in a quartz cuvette using a Malvern ZEN1600 instrument (Malvern Instruments Ltd, Worcestershire, United Kingdom) operated using the Zetasizer Nano software. The samples were first measured at low temperature (measurement 1), then the temperature was raised to 7 °C above the denaturation temperature and equilibrated for 5 minutes before the DLS (measurement 2) was started. After this, the temperature was lowered to the starting temperature and after 5 minutes of equilibration DLS was measured again (measurement 3). The estimated molecular weight (in kDa) and the %-polydispersity based on size distribution are given for experiments 1 and 3 in the Table above. A plot with the size-distribution before and after heating at elevated temperature is also shown.
