# Supplementary material for: Mechanism of Protein Kinetic Stabilization by Engineered Disulfide Crosslinks
Source: PLoS One. 2013 Jul 30;8(7):e70013. doi: 10.1371/journal.pone.0070013 (PMC3728334; doi:10.1371/journal.pone.0070013)
Supplement: Figure S2 — Rate constants obtained with total protein concentration of 0.25 and 1 mg/mL. (PDF) [file pone.0070013.s002.pdf]

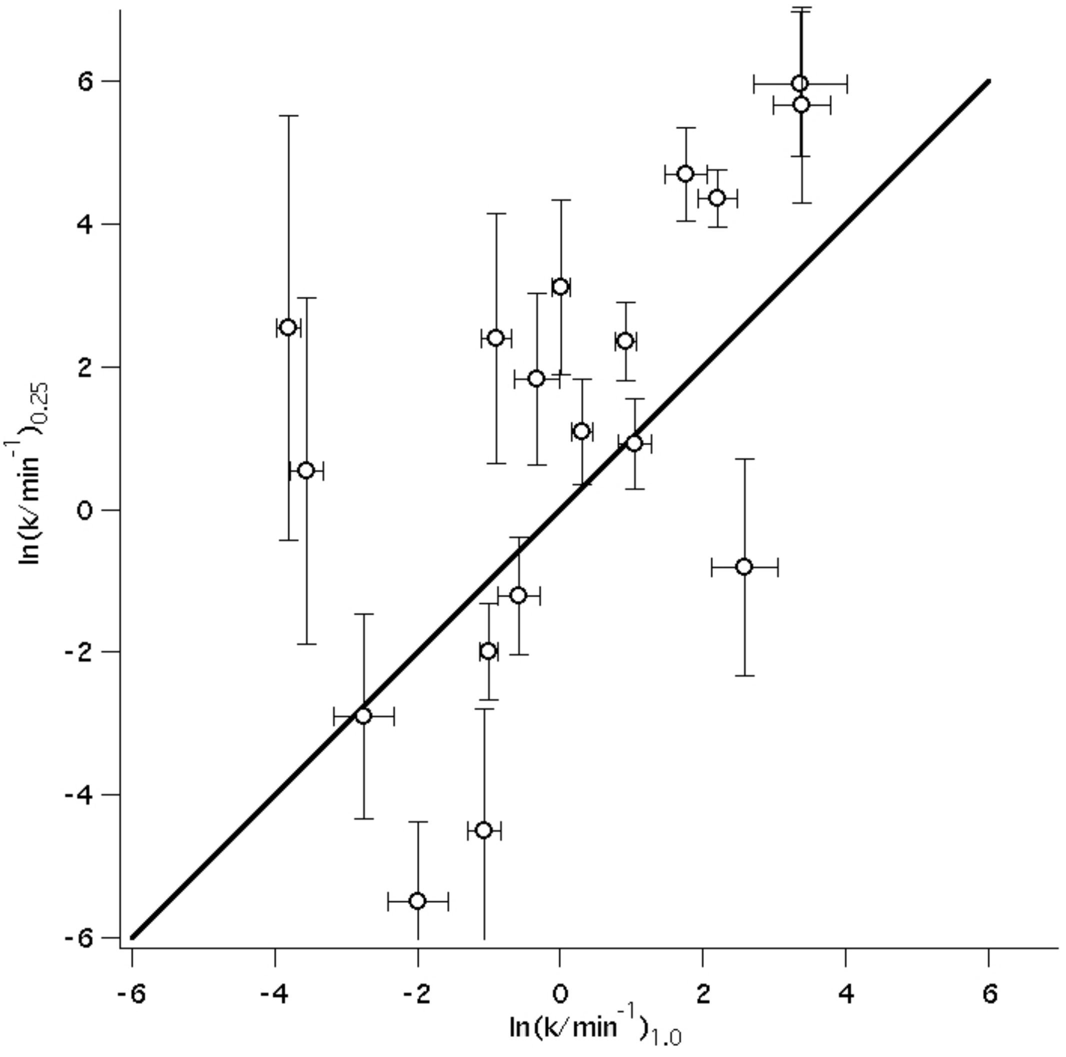

Figure S2. Rate constants obtained with total protein concentration of 0.25 and 1 mg/mL. The phenomenological adequacy of equation 1 (main text) can break down at low protein concentrations, as suggested by the weak correlation of the rate constants obtained at 0.25 and 1 mg/mL.
