# Supplementary material for: Mechanism of Protein Kinetic Stabilization by Engineered Disulfide Crosslinks
Source: PLoS One. 2013 Jul 30;8(7):e70013. doi: 10.1371/journal.pone.0070013 (PMC3728334; doi:10.1371/journal.pone.0070013)
Supplement: Figure S3 — Temperature dependence of the time scale (τ1/2) for the irreversible denaturation of wild type phytase and variants at a protein concentration of 1 mg/mL. (PDF) [file pone.0070013.s003.pdf]

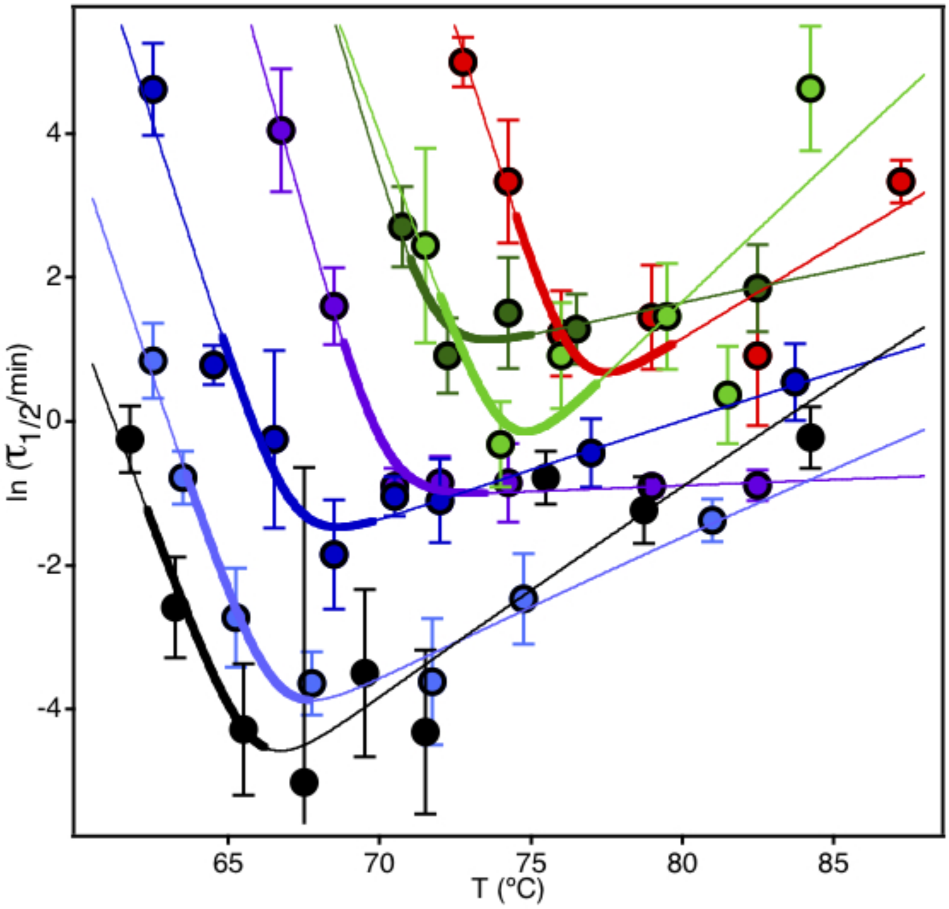

Figure S3. Temperature dependence of the time scale ($\tau_{1/2}$) for the irreversible denaturation of wild type phytase and variants at a protein concentration of 1 mg/mL. The continuous lines represent the best fits of equation 3. Thicker lines indicate the temperature range of denaturation transition as seen by DSC (Figure 1A, main text). The colors of the lines and data points refer to the variant, as specified in panel A of Figure 1 (main text).
