# Supplementary material for: Mechanism of Protein Kinetic Stabilization by Engineered Disulfide Crosslinks
Source: PLoS One. 2013 Jul 30;8(7):e70013. doi: 10.1371/journal.pone.0070013 (PMC3728334; doi:10.1371/journal.pone.0070013)
Supplement: Text S1 — Experimental section. (DOC) [file pone.0070013.s004.doc]

**SUPPORTING INFORMATION S1. EXPERIMENTAL SECTION**

**Molecular Dynamics simulations**. The structure of the *C. braakii* ATCC 51113 phytase was built by homology modeling using the program nest from the jackal package (Prof. Barry Honig, Columbia University (http://wiki.c2b2.columbia.edu/honiglab_public/index.php/Software:Jackal), using the structure of the *E. coli* AppA phytase (Protein Data Bank id.: 1DKO) as template. Molecular dynamics (MD) simulations in aqueous solution were carried at four different temperatures (300, 350, 400 and 500 K) starting from the resulting model. The CHARMM22 force field was used along with the MD engine NAMD. All simulations were run for at least for 2 nano seconds (ns) except that carried out at 500 K which was prolonged to 10 ns. Simulation snapshots were saved every picosecond. The long-range interactions were switched from 8 to 12 Å and the distance between pairs for inclusion in pair lists was 13.5 Å. A new pair list was generated every five integration steps. Hydrogen bonds were kept rigid using the SHAKE algorithm for the protein and the non-iterative SETTLE algorithm for water; a 2 femtosecond time integration step was consequently used. The microcanonical (NVE) ensemble was sampled. A cubic simulation box of side 100 Å was used. The density of the water was adjusted for each temperature.

**Phytase expression in *Aspergillus oryzae*.** The constructs comprising the *C. braakii* phytase variant genes in the examples were used to construct expression vectors for *Aspergillus*. The *Aspergillus* expression vectors consist of an expression cassette based on the *A. niger* neutral amylase II promoter fused to the *A. nidulans* triose phosphate isomerase non translated leader sequence (Pna2/tpi) and the *A. niger* amyloglycosidase terminator (Tamg). Also present on the plasmid was the *Aspergillus* selective marker pyrG from *A. nidulans* enabling growth on miminal media for an *Aspergillus* which is pyrG minus. The expression plasmids for phytase variants were transformed into *Aspergillus* as described. For each of the constructs 4-6 strains were isolated, purified and cultivated in microtiterplates. Expression was determined using a *p*-nitrophenyl phosphate (Sigma, N-9389) substrate and the best producing strain was fermented in shake flasks.

**Phytase purification.** The fermentation supernatant with the variant was filtered through a Fast PES Bottle top filter with a 0.22 μm cut-off. The resulting solution was diluted with water to double its volume and the pH adjusted to 4.5 with acetic acid. After pretreatment the variant was purified by chromatography on S-Sepharose, approximately 30 ml in a XK26 column, using as buffer A 50 mM sodium acetate pH 4.5, and as buffer B 50 mM sodium acetate + 1 M NaCl pH 4.5. The fractions from the column were analyzed for activity using *p*-nitrophenyl phosphate (Sigma, N-9389) and fractions with activity were pooled. Finally, deglycosylation was performed using endo-H. The molecular weight, as estimated from SDS-PAGE, was approximately 45-50 kDa and the purity was > 95%.

**Phytase activity assays.** Activity was determined with an end-point assay measuring total released phosphate from a sodium phytate solution adapted from the method of Engelsen et al. to the microtitre well format. We made sure that all our activity determinations were carried within the linear region of the activity versus phytase concentration dependence. Most assays were performed in 50 mM sodium acetate pH 5.5 and 37°C. However, we also determined the activity/pH profile (Figure 3C in the main text) at 37°C in the pH range of 2.0 to 7.5 (in 0.5 pH-unit steps in a buffer cocktail) and the activity/temperature profile (50 mM sodium acetate pH 5.5) in the temperature range 20-90°C (Figure 3B in the main text).

**X-ray crystallography**. Crystallization screening was performed at room temperature with commercially available screens using sitting-drop vapour-diffusion. Drops were set up employing a *Mosquito Crystal* liquid handling robot (TTP LabTeck, UK) with 150 nl protein solution plus 150 nl reservoir solution in 96-well format plates (MRC 2-well crystallization microplate, Swissci, Switzerland) equilibrated against 54 µl reservoir solution. Extensive screening of the wild-type *C. braakii* phytase over a long period failed to produce diffraction quality crystals. Screening subsequently focused on the series of engineered disulphide variants. This led to the successful crystallization of the S3 variant, with a disulfide bridge introduced between residues 141 and 199.

Two screen conditions (F3 of York’s Hampton I & II screen: 4.0 M sodium formate and C4 of Hampton’s Salt Rx screen: 3.5 M sodium formate + 0.1 M Bis-Tris propane pH 7.0) produced crystals which diffracted to around 3 Å in-house using a Rigaku Micromax-007 X-ray generator (Cu Kα, λ = 1.54179 Å) equipped with a MAR345 image plate detector (Marresearch GmbH, Germany). Data were collected from a crystal grown from 4.0 M sodium formate at beamline ID14-1 of the ESRF to a resolution of 2.3 Å and processed in space group *P*3_1_21 (a = b = 121.51 Å, c = 129.11 Å). X-ray data were processed using programs from the CCP*4* suite. The images were integrated with MOSFLM and then scaled with *SCALA* ). Molecular replacement was successfully carried out using *MOLREP* with the *H. alvei* phytase (PDB code 4ARS) as a search model. The residues were rebuilt using *BUCCANEER* . The structures were completed using iterative cycles of *COOT* and *REFMAC5* . Refinement converged to an R_fac_ of 19.78% and an R_free_ of 24.45%, Table 1. Structure validation with PROCHECK showed 92.5% of residues in the most favored and 7.5% in the additional allowed regions of the Ramachandran plot. There are two independent protein monomers in the asymmetric unit. For chain A there was good electron density for residues 6-116, 120-138, 14-179, 186-201, 208-410, and for chain B residues 5-201, 208-222, 224-411. The disordered residues lie at the termini or in loops at some distance from the active site. The final model includes 29 formate molecules, 9 Cl^-^ ions and 574 water molecules. The Cl^-^ ions are not visible in the anomalous difference map but are modeled as such as the electron density for water molecules in these positions is unsatisfactory.

Structural superposition was carried out with the SSM algorithm. The atomic coordinates and structure factors have been deposited in the Protein Data Bank, www.ebi.ac.uk (PDB ID code 3zhc).

**Differential scanning calorimetry**. Experiments were performed using a VP-Capillary DSC from MICROCAL GE Healthcare at a scan-rate of 200 K/h and a protein concentration of 0.5 mg/mL. Protein samples were prepared by exhaustive dialysis against the buffer (20 mM sodium acetate pH 4.5). For several variants, additional experiments were performed at 60, 120 and 200 K/h to check the presence of scan-rate effect; and at 0.2, 0.5 and 1 mg/mL to check the presence of concentration effect. In all measurements, the buffer from the final dialysis step was used in the reference cell of the calorimeter. Several buffer-buffer baselines were obtained before each protein run for proper equilibration of the calorimeter.

**Thermal inactivation experiments**. 15 μL protein solution aliquots (20 mM sodium acetate buffer, pH 4.5) were transferred to 0.2 mL PCR eppendorf tubes. At time zero, tubes were immersed in a water bath set at the desired experiment temperature. After 2, 4, 8, 16, 32, 64, 128 and 256 minutes, tubes were transferred to ice/water to stop the irreversible denaturation process. After about 300 min, the protein solutions in the tubes were diluted to a final concentration of 0.02 mg/ml and phytase activity assays performed at 37 ºC as described above. All activity determinations were performed within the linear-response range of the activity/concentration profile. The value of the activity at time zero was determined as described above, except that the corresponding tube was not immersed in the high-temperature water bath, but kept at room temperature. Thermal inactivation experiments were carried for all variants at different temperatures and, typically, at a total protein concentration of 0.5 mg/ml. To test the possibility of a protein concentration dependence of the inactivation kinetics, additional experiments were performed at 0.25 and 1.0 mg/ml.

**References**

1. Lim D, Golovan S, Forsberg CW, Jia Z (2000) Crystal structures of Escherichia coli phytase and its complex with phytate. Nature structural biology 7: 108-113.

2. A. D. MacKerell J, D. Bashford, M., R. L. Dunbrack, Jr., J. D. Evanseck, M. J. Field, S. Fischer, J. Gao, H. Guo, S. Ha, D. Joseph-McCarthy, L. Kuchnir, K. Kuczera, F. T. K. Lau, C. Mattos, S. Michnick, T. Ngo, D. T. Nguyen, B. Prodhom, W. E. Reiher, B. Roux, M. Schlenkrich, J. C. Smith, R. Stote, J. Straub, M. Watanabe, J. Wiórkiewicz-Kuczera, D. Yin, and M. Karplus (1998) All-Atom Empirical Potential for Molecular Modeling and Dynamics Studies of Proteins. J Phys Chem B 102: 3586-3616.

3. Phillips JC, Braun R, Wang W, Gumbart J, Tajkhorshid E, et al. (2005) Scalable molecular dynamics with NAMD. Journal of computational chemistry 26: 1781-1802.

4. Ryckaert J, Ciccotti, G, Berendsen, HJC (1977) Numerical integration of the cartesian equations of motion of a system with constraints: molecular dynamics of n-alkanes. Journal of Computational Physics 23: 327-341.

5. Miyamoto SK, PA (1992) Settle: An analytical version of the SHAKE and RATTLE algorithm for rigid water models. Journal of Computational Chemistry 13: 952-962.

6. Kell G (1967) Precise representation of volume properties of water at one atmosphere. J Chem Eng Data 12: 66-69.

7. Lassen SF, Breinholt J, Ostergaard PR, Brugger R, Bischoff A, et al. (2001) Expression, gene cloning, and characterization of five novel phytases from four basidiomycete fungi: Peniophora lycii, Agrocybe pediades, a Ceriporia sp., and Trametes pubescens. Applied and environmental microbiology 67: 4701-4707.

8. Engelen AJ, van der Heeft FC, Randsdorp PH, Smit EL (1994) Simple and rapid determination of phytase activity. Journal of AOAC International 77: 760-764.

9. Winn MD, Ballard CC, Cowtan KD, Dodson EJ, Emsley P, et al. (2011) Overview of the CCP4 suite and current developments. Acta crystallographica Section D, Biological crystallography 67: 235-242.

10. Leslie AGW (1992) Recent changes to the MOSFLM package for processing film and image plate data. Joint CCP4 and ESF-EACMB newsletter on protein crystallography: Daresbury Laboratory, Warrington, UK.

11. Kabsch W (1988) Evaluation of single-crystal X-ray diffraction data from a position-sensitive detector. Journal of Applied Crystallography 21: 916-924.

12. Evans P (2006) Scaling and assessment of data quality. Acta Crystallographica Section D 62: 72-82.

13. Vagin A, Teplyakov A (1997) MOLREP: an automated program for molecular replacement. J Appl Cryst **30**: 1022-1025.

14. Cowtan K (2006) The Buccaneer software for automated model building. 1. Tracing protein chains. Acta Crystallographica Section D-Biological Crystallography 62: 1002-1011.

15. Emsley P, Cowtan K (2004) Coot: model-building tools for molecular graphics. Acta Crystallogr D60: 2126-2132.

16. Murshudov GN, Vagin AA, Dodson EJ (1997) Refinement of macromolecular structures by the maximum likelihood method. Acta Cryst D 53: 240-255.

17. Morris AL, MacArthur MW, Hutchinson EG, Thornton JM (1992) Stereochemical quality of protein structure coordinates. Proteins 12: 345-364.

18. Krissinel E, Henrick K (2004) Secondary-structure matching (SSM), a new tool for fast protein structure alignment in three dimensions. Acta crystallographica Section D, Biological crystallography 60: 2256-2268.
