# Supplementary material for: Mechanism of Protein Kinetic Stabilization by Engineered Disulfide Crosslinks
Source: PLoS One. 2013 Jul 30;8(7):e70013. doi: 10.1371/journal.pone.0070013 (PMC3728334; doi:10.1371/journal.pone.0070013)
Supplement: Text S2 — Theoretical analysis of a thermal inactivation model involving a critical, lowly-populated intermediate species. (DOC) [file pone.0070013.s005.doc]

**SUPPORTING INFORMATION S2. THEORETICAL ANALYSIS OF A THERMAL INACTIVATION MODEL INVOLVING A CRITICAL, LOWLY-POPULATED INTERMEDIATE SPECIES**

We analyzed the experimental activity/time profiles for the inactivation of phytase in terms of a simple, phenomenological, n-order rate equation:

(S1)

where n is the order of reaction and (P) is the total concentration of protein that is not irreversibly denatured at a time t. In terms of the model of Figure 8A in the main text (P)=(N)+(I)+(U), although we will assume that the population of I is always very low and, therefore, (P)≅(N)+(U). Straightforward integration of equation S1 (as described in Physical Chemistry textbooks) leads to equation 1 in the main text. Fits of equation 1 to the experimental inactivation profiles (Figure 6 in the main text) yields values of the rate constant k which can be used to calculate values for the time at which the activity (proportional to (P)) falls to half the initial value:

(S2)

where C is the total protein concentration (including irreversibly denatured protein). Equation S2 is of course equivalent to equation 2 in the main text.

The denaturation model we analyze here (Figure 8A in the main text) suggests, on the other hand, a rate equation of the form:

(S3)

which can be interpreted as suggesting that a species with m intermediate molecules is critical for the irreversible denaturation process and that this species “recruits” n-m molecules (either native, unfolded or intermediate) to form the kinetically relevant transition state with n molecules. Defining X_I_ as the fraction of P (protein that is not irreversibly denatured) present as intermediate I, we have:

(S4)

and, therefore, equation S3 is compatible with the phenomenological rate equation (equation S1) provided that,

(S5)

We further assume that all the temperature and disulfide-crosslink effects on the rate of irreversible denaturation reflect the amount of intermediate I (i.e., X_I_ in equation S4). That is, we assume that k_0_ is strictly a constant so that we can write lnτ_1/2_ (equation S2) as,

(S6)

where B is constant (for a given total protein concentration):

(S7)

To obtain an expression for X_I_, we assume equilibrium between N, I and U to be established and use standard binding polynomial formalism to write X_I_ as,

(S8)

where K_I_ and K_U_ are the equilibrium constants for the N⇔I and N⇔U processes, and in the second equality we have assumed that K_I_<<1+K_U_. That is, we assume that the concentration of I is always low compared with the total concentration of protein that is not irreversibly denatured ((I)<<(N)+(U) and X_I_<<1).

The temperature dependencies of K_U_ and K_I_ can be described by the integrated van’t Hoff equation:

(S9)

(S10)

where T_0_ is the equilibrium denaturation temperature (by definition, the temperature at which the unfolding equilibrium constant, K_U_, equals unity), K_I_^0^ is the value of K_I_ at the temperature T_0_, and ΔH_U_ and ΔH_I_ are the enthalpies of U and I relative to the native state. Substituting equations S9 and S10 into equation S8 and using the resulting equation in equation S6, we easily arrive at:

(S11)

which is equation 3 in the main text with the constant A defined as:

(S12)

It must be noted that equations S9, S10 and, consequently, S11 implicitly assume that the temperature dependencies of ΔH_I_ and ΔH_U_ can be neglected within the comparatively narrow range of each lnτ_1/2_ vs. T profile and therefore the ΔH_I_ and ΔH_U_ values in equation S11 are to be assigned at the temperature T_0_. Also, it would appear that the ΔH_U_ to be used in equation S11 could be derived from the experimental DSC transitions as the calorimetric enthalpy values (values of the area under the transitions). However, the phytase experimental DSC profiles are distorted by irreversible denaturation (i.e., the thermal denaturation process leads to a final irreversible-denatured state, which is likely aggregated protein) while ΔH_U_ is the unfolding enthalpy change associated to the process N→U. In order to contribute to robust fits based on equation S11, we deemed convenient to estimate ΔH_U_ from well-established structure-energetics relationships and we used in fact the ΔH_U_ values calculated from the dependencies of the energetic parameters with the number of residues reported by Robertson and Murphy. This leads to the temperature-dependent ΔH_U_ values shown as a blue line in Figure 7C of the main text. Actually, our fitting procedure employed the ΔH_U_ value corresponding to the temperature T_0_ considered at each stage of the fitting process. Since the value of ΔH_U_ has been determined by structure-energetic relationships, the fits of equation S11 to the experimental lnτ_1/2_ vs. T profiles involve only three fitting parameters (A, T_0_ and ΔH_I_), provided of course that a decision is made regarding the value to be used for the reaction order for I (m). Our approach to the determination of the m value involved performing fits to all profiles assuming different values of m (1, 2, 3, 4…) and plotting the sum of a measure goodness of fit (sum of least-squares deviations) for all the fits versus the m value. This plot is shown as an inset in Figure 7B of the main text and strongly supports a value of unity for m. Furthermore, the values of the equilibrium denaturation temperature (T_0_) obtained with m=1 are in excellent agreement with the transition temperatures from DSC once the kinetic distortion in the latter values is taken into account (see Figure 5E, main text). This agreement, together with the fact that good fits are obtained using unfolding enthalpy values (ΔH_U_) derived from established structure-energetics relationships, strongly supports the adequacy of the model embodied in equation S11 (equation 3 in the main text).

The fits of equation S11 to the experimental lnτ_1/2_ vs. T profiles yield values for the enthalpy of the lowly-populated intermediate relative to the native state (ΔH_I_ values in Figure 7C). However, in order to achieve a more complete energetic description of I, it is desirable to have some estimate of its free energy relative to the native state (i.e., the ΔG_I_ value). ΔG_I_ for a given phytase variant at a temperature equal to its T_0_ value is given by,

(S13)

but it cannot be calculated because K_I_^0^ is not available from experiment (i.e., it appears combined with other paramentes in the constant A: equation S12). Nevertheless, we can calculate from the values of A, T_0_ and m (=1) a suitable metric of the free energy of I (ΔG_I_*) as,

(S14)

where, in the second equality, we have used equation S12. The rationale behind this procedure is that, since B is taken to a constant for a given total protein concentration, it will cancel out upon carrying differences in the ΔG_I_* metric. Accordingly, the ΔG_I_* difference between a variant and the wild type should actually equal the effect of the engineered disulfide bridges in the variant on the free energy of the intermediate relative to the native state (the ΔΔG_I_ value). However, the value of the ΔG_I_* metric calculated as ART_0_/m are assigned to the temperature T_0_ and different variants display different T_0_ values. Therefore, before the “variant minus wild type” subtraction can be performed, the ΔG_I_* values must be referred to a common temperature. This can be done using well-known thermodynamics (the Gibbs-Helmholtz equation):

(S15)

and integrating (with ΔH_I_(T)=ΔH_I_+ΔC_P,I_·(T-T_0_)) and using S14, we arrive at:

(S16)

where ΔH_I_ is the value for the enthalpy of the intermediate determined from the fitting of equation S11 to the experimental lnτ_1/2_ vs. T profiles (and, therefore, corresponding to T_0_) and ΔC_P,I_ is the heat capacity of the intermediate relative to the native state. A similar Gibbs-Helmholtz analysis leads to free energy of the unfolded state (relative to the native state) at the temperature T´:

(S17)

where ΔH_U_ is the value of the enthalpy of U (relative to N) at the temperature T_0_ and ΔC_P_ is the unfolding heat capacity change (the heat capacity of U relative to N). The ΔC_P_ value to be used in equation S17 was derived from the dependencies of the protein unfolding energetic parameters with the number of residues reported by Robertson and Murphy. ΔC_P,I_ (to be used in equation S16) was assumed equal to ΔC_P_ as the enthalpy values (Figure 7C) suggest a highly unfolded intermediate. It is important to note, in any case, that the terms in heat capacity in equations S16 and S17 are minor and the calculation is robust to the values chosen for the heat capacity changes. Actually, assuming values of zero for the heat capacity changes yields essentially identical free energies.

Equations S16 and S17 were used to estimate the free energies of I and U for all the variants studied at a common temperature of T´=70 ºC (i.e., approximately in the middle of the experimental range to avoid long extrapolations) and “variant minus wild-type” subtractions were performed to obtain the corresponding free-energy effects of the engineered disulfide bridges:

(S18)

(S19)

which could be used to perform a classical φ-value calculation of the structure of the intermediate around the mutation sites:

(S20)

We have chosen instead to make a plot of ΔΔG_I_ versus ΔΔG_U_ (Figure 7D). The slope of this plot indicates an average φ-value around unity, which is consistent with a highly unfolded intermediate.

**References**

1. Robertson AD, Murphy KP (1997) Protein Structure and the Energetics of Protein Stability. Chemical reviews 97: 1251-1268.
